# Safety and therapeutic potential evaluation of Cefotaxime plus Polymyxin B against polymyxin-carbapenem resistant *Klebsiella pneumoniae* in a murine model

**DOI:** 10.1371/journal.pone.0339990

**Published:** 2026-01-05

**Authors:** Mariana Carvalho Sturaro, Nathalia da Silva Damaceno, Gleyce Hellen de Almeida de Souza, José Eduardo Souza Echeverria, Ediane Bortolotte Cornelius, Luccas Pereira Pires, Pedro Vinícius Dias Bassetto Silva, Bárbara Maria Cristaldo Gomes, Thiago Leite Fraga, Osmar Nascimento Silva, Luana Rossato, Simone Simionatto

**Affiliations:** 1 Laboratory of Research in Health Science, Federal University of Grande Dourados, Dourados, Mato Grosso do Sul, Brazil; 2 University Center of Grande Dourados – UNIGRAN, Dourados, Mato Grosso do Sul, Brazil; 3 Evangelical University of Goiás, Anápolis, Goiás, Brazil; Rivers State University, NIGERIA

## Abstract

**Objectives:**

This study aimed to evaluate the toxicity and antibacterial efficacy of Cefotaxime/Polymyxin B combination (CTX/PMB) against a polymyxin-carbapenem-resistant (PC-R) *Klebsiella pneumoniae* strain, using a mice model.

**Methods and results:**

A single-dose toxicity assay was conducted in BALB/c mice, divided into control and CTX/PMB-treated groups receiving low, medium, or high CTX doses. Body weight, food, and water intake were monitored for 14 days. After euthanasia, organ weights and plasma biochemical markers were analyzed. Medium- and high-dose groups maintained stable weight and intake. High-dose mice exhibited reduced right kidney and liver weights and elevated urea levels. Creatinine was at the upper limit in all groups, including one control mouse. For antimicrobial efficacy, BALB/c neutropenic mice infected with PC-R *K. pneumoniae* K18 were assigned to naïve, mock-treated, CTX, PMB, or CTX/PMB groups. Treatments were given every 12 h, and after 24 h, blood was collected to quantify bacterial load. CTX/PMB significantly reduced blood bacterial load and improved clinical condition compared to other groups.

**Conclusion:**

CTX/PMB showed therapeutic potential in treating PC-R *K. pneumoniae*. However higher CTX doses may potentiate PMB-associated toxicity. These findings encourage further investigation in advanced preclinical models and clinical settings to fully elucidate CTX/PMB therapeutic potential and optimize dosing regimens.

## 1. Introduction

The global rise in antimicrobial resistance (AMR) poses a significant public health challenge, accounting for about 9% of annual deaths worldwide and significantly contributing to healthcare-associated morbidity and mortality [1]. *Klebsiella pneumoniae,* a gram-negative pathogen, is one of the six major causes of hard-to-treat hospital-acquired infections; it can rapidly develop resistance to multiple classes of antibiotics [2,3]. In response, the World Health Organization have prioritized the development of novel antimicrobial strategies to address this escalating threat [4,5].

However, developing new antibiotics is a long and costly process, often taking 10–15 years, which poses a challenge given the rapid pace of AMR. In this context, antibiotic combination therapy offers a suitable alternative, since the drugs involved are well-known [6]. When used synergistically, combination treatments can broaden the antibacterial spectrum, increase bacterial killing, decrease the chances of developing resistance, and lower the required doses of individual drugs, thereby minimizing toxicity [7–9]. They can also target multiple bacterial pathways simultaneously, making it more difficult for pathogens to adapt and survive; thus, they are particularly effective against multidrug-resistant (MDR) strains [10].

Cefotaxime (CTX), a third-generation cephalosporin, is widely used as a broad-spectrum antibiotic [11]. Although β-lactam resistance mechanisms, especially those driven by plasmid-mediated extended-spectrum β-lactamases (ESBLs), have emerged, CTX remains effective against many bacterial strains in clinical practice [12,13]. To address these challenges and improve treatment outcomes, combination strategies involving CTX and β-lactamase inhibitors (e.g., sulbactam) [14], or other synergistic antimicrobials (e.g., rifampicin, ciprofloxacin, delafloxacin) are being explored to enhance efficacy against resistant strains [15,16].

A synergistic interaction between CTX and polymyxin B (PMB) was observed in a recent study against a multidrug-resistant, polymyxin-carbapenem resistant (PC-R) *K. pneumoniae* strain [17]. While the toxicity profiles of CTX and PMB mono-therapies are available, their combined use, denoted here as CTX/PMB, has not been thoroughly investigated. Preclinical studies are needed to ensure the safety and efficacy of new treatment strategies, serving as an important step toward real-world application [18]. Therefore, this study evaluated CTX/PMB in a mouse model by assessing both potential toxicity indices by performing an extended single-dose assay and determining antimicrobial activity against PC-R *K. pneumoniae* in a mammalian system.

## 2. Material and methods

### 2.1 Chemicals

CTX sodium salt (Lot #0000334303) and PMB solution at a concentration of 1 mg/mL (Lot #BCCG2613) were purchased from Sigma-Aldrich. CTX was prepared according to the manufacturer’s instructions, being diluted in saline solution for use in animal experiments.

### 2.2 Animals

BALB/c mice (8–12 weeks old, 24–28 g) were obtained from the Central Animal Facility of the Federal University of Mato Grosso do Sul. Prior to experimentation, the animals underwent a one-week acclimatization period. The mice were housed in polypropylene cages under controlled conditions: temperature (22 ± 3 °C), humidity (40–60%), and a 12 h/12 h light-dark cycle. Standard commercial feed and water were provided *ad libitum*. All procedures were performed between September and November 2024 in accordance with guidelines established by the National Council for the Control of Animal Experimentation (CONCEA). Further, the study protocol was reviewed and approved by the Animal Ethics Committees of the Federal University of Grande Dourados (protocol no. 23018) and the University Center of Grande Dourados – UNIGRAN (protocol no. 080/18). Manipulations were performed by personnel who had undergone prior training in rodent handling, under the guidance of senior staff with extensive experience in mouse experimentation. Humane endpoints were predefined in accordance with institutional animal care standards, including criteria such as >20% body weight loss, persistent immobility, inability to access food or water, or signs of severe pain or distress. Throughout the study, no animals met these humane endpoint criteria.

### 2.3 Extended single-dose toxicity assay

An extended single-dose toxicity assay was conducted to evaluate the toxicity of CTX/PMB [19]. Briefly, the animals were randomly divided into four groups, each consisting of six mice. The mice were administered a single intraperitoneal (i.p.) injection of the following treatments: (1) Control: vehicle only (saline solution); (2) Low-dose: CTX/PMB with CTX at 2 g; (3) Medium-dose: CTX/PMB with CTX at 4 g; (4) High-dose: CTX/PMB with CTX at 8 g (Table 1). The low CTX dose was based on standard human posology (assuming a 70 kg adult) for uncomplicated infections [20] and adapted for mice using body weight scaling. The medium and high doses corresponded to two times and four times the standard dose, respectively. The PMB concentration was fixed at 2 mg/kg across all combination groups, corresponding to its standard posology [21].

**Table 1:** Treatment groups and survival rates in the extended single-dose toxicity assay for CTX/PMB.

Group	Treatment dosage		Survival (%)
	Cefotaxime g/day*	Polymyxin B mg/kg/day	
Control	0	0	100
Low-dose	2	2	100
Mid-dose	4	2	100
High-dose	8	2	100

*Doses were derived from human therapeutic regimens (70 kg reference weight) and translated to mice using body weight-based scaling.

#### 2.3.1 Animal monitoring.

After treatment, the mice were monitored daily for 14 days to assess their general condition, including behavior, appearance, physiological function, presence of convulsions, pruritus, ataxia, and body weight [22]. Food consumption was evaluated by measuring the daily reduction from a standard 0.2 kg portion. Water consumption was evaluated using the same approach, with an initial volume of 0.6 L of water provided per cage. Body weight, food intake, and water consumption over time were analyzed by calculating the area under the curve (AUC) using the trapezoidal rule [23].

#### 2.3.2 Blood collection and organ weight measurement.

After monitoring for 14 days, the mice were anesthetized via i.p. injection of a combination of xylazine (10 mg/kg) and ketamine (60 mg/kg). Euthanasia was performed by exsanguination under deep anesthesia. Blood samples collected during this procedure were analyzed for biochemical parameters. Then, the mice were dissected following standard pathological procedures. The liver, spleen, and both kidneys were excised and weighed [24].

#### 2.3.3 Biochemical analyses.

For biochemical analyses, plasma was obtained by centrifuging the blood sample collected in tubes, without anticoagulant, at the exsanguination procedure [25]. An automated clinical chemistry analyzer, Mindray BS-120 (Shenzhen Mindray Bio-Medical Electronics Co., Ltd., Shenzhen, China), was used to perform biochemical serum analyses. The parameters evaluated included creatinine, urea, alanine aminotransferase, alkaline phosphatase, aspartate aminotransferase, total bilirubin, direct bilirubin, and indirect bilirubin. All analyses were conducted under standardized laboratory conditions, and the results were interpreted based on reference values.

### 2.4 Infection model

#### 2.4.1 Bacterial strain and culture conditions.

To evaluate the antibacterial activity of CTX/PMB, an infection model was established using the PC-R *K. pneumoniae* K18 strain, which was previously described exhibiting resistance to carbapenems, β-lactams, and polymyxins [26]. For the experiment, the isolate was initially cultured in brain heart infusion (BHI) broth at 37 °C for 24 h. Following this incubation, the culture was streaked onto BHI agar and incubated under the same conditions at 37 °C for 24 h. This protocol ensured the optimal growth and viability of PC-R *K. pneumoniae* K18 for the subsequent assay.

#### 2.4.2 *In vivo* antibacterial activity evaluation.

Briefly, dexamethasone (20 mg/kg) was administered via the i.p. route to the mice 24 h before the experiment to induce immunosuppression and increase model sensitivity. All neutropenic mice, except those in the naïve group, were administered an i.p. injection of 0.2 mL of bacterial suspension containing 3 × 10^8^ CFU/mL, prepared in 0.9% saline solution [27]. The mice were randomly assigned to five groups as follows: (1) naïve: uninfected control (n = 4); (2) mock-treated: infected but untreated control (n = 5); (3) CTX: received CTX 2 g/kg/day (dose adjusted to mouse body weight) i.p. (n = 4); (4) PMB: received PMB 1 mg/kg/day i.p. (n = 4); and (5) CTX/PMB: received both CTX (2 g/day) and PMB (1 mg/kg/day) i.p (n = 4). The CTX dose was selected based on the results of the toxicity assay, whereas the PMB dose was determined according to its pharmacokinetic profile; specifically, the plasma concentration required over time to reach its effective *in vitro* dose in an MDR *K. pneumoniae* infection model [28]. Animals were monitored every 6 h for well-being and mortality. After 24 h, the mice were anesthetized via i.p. injection of xylazine (10 mg/kg) and ketamine (60 mg/kg). Euthanasia was performed by exsanguination under deep anesthesia. Blood samples collected at this stage were plated on BHI agar containing meropenem to selectively recover resistant bacteria. After 24 h of incubation at 37 °C, bacterial colonies were counted and expressed as log_10_ CFU/mL, with a lower detection limit set at 1.69 log_10_ CFU/mL.

### 2.5 Statistical analyses

Statistical analyses were performed using One-way analysis of variance (ANOVA) followed by Tukey’s test for group comparisons. Survival of infected mice was evaluated using Kaplan–Meier survival curves. Differences were considered statistically significant at p < 0.05. All analyses were conducted using GraphPad Prism version 8.0 (GraphPad Software, San Diego, CA, USA).

## 3. Results

### 3.1 Extended single-dose toxicity assay

#### 3.1.1 Animals general condition.

Although CTX and PMB have known safety profiles, the effects of their combination are not known. In the groups treated with CTX/PMB, which comprised three different doses of CTX along with a fixed dose of PMB, no deaths or visible abnormalities were recorded, as was the control group (vehicle only). All treated mice showed 100% survival (Table 1). Throughout 14 days of monitoring, the animals remained socially active, with no apparent changes in behavior or general condition.

#### 3.1.2 Animals body weight and food consumption.

**Fig 1:**
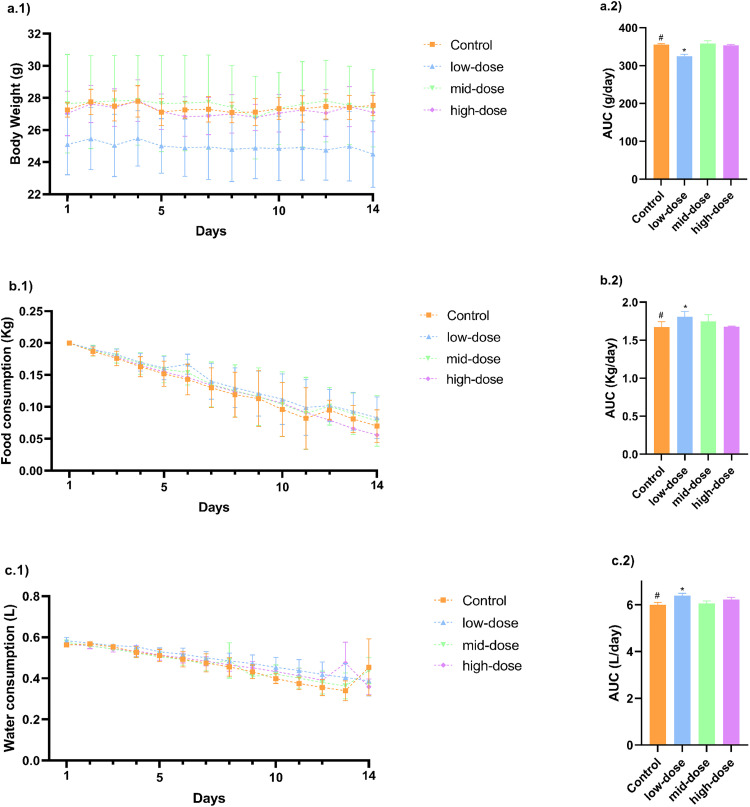
Mice body weight (A.1) was monitored over 14 days following a single dose of CTX/PMB at three different CTX concentrations (low, mid, and high) or vehicle (control). The area under the weight curves was calculated for each group **(A.2)**. Food consumption was recorded daily for all groups **(B.1)**, and corresponding areas under the food consumption curves were obtained **(B.2)**. Water consumption was monitored over the same period **(C.1)**, and the area under the curves was calculated for each group **(C.2)**. Area under the curve (AUC) values were analyzed for statistical significance using one-way ANOVA, with p < 0.05 considered significant.

Throughout 14 days of monitoring, the medium-dose and high-dose groups maintained stable body weights, showing no significant increasing trends, similar to those of the control group. However, the low-dose group presented a lower average body weight from the start of the experiment, with only a slight reduction of 0.5 g by the final day (Fig 1A.1). This difference was statistically significant compared to the control group, as determined by the analysis of the weight/time AUC (Fig 1A.2).

Food consumption was assessed by measuring the reduction in a standard portion over time; thus, the area under the plotted curves was inversely proportional to actual intake. All groups consumed similar amounts of food throughout the experiment (Fig 1B.1). Water consumption was evaluated using the same method, with comparable results recorded across groups (Fig 1C.1). The low-dose group presented slightly lower food and water intake (as shown in AUC analyses), which reflected the lower median body weight of the mice in this group (Fig 1B.2 and 1C.2).

#### 3.1.3 Mice organ weights.

Organ weights were measured to assess potential effects of single-dose CTX/PMB administration (Table 2). The low-dose group presented the lowest values across all organs assessed, which was probably attributable to their overall lower body weight. Medium-dose group did not have any significant difference in organs weight. However, compared to the control (vehicle only), the high-dose group presented statistically significant reductions (p < 0.05) in the right kidney and liver.

**Table 2:** Mice organ weights 14 days after receiving a single dose of CTX/PMB or vehicle.

Organ	Control	Cefotaxime/Polymyxin B		
		Low-dose	Mid-dose	High-dose
Spleen	0.126 ± 0.007	0.113 ± 0.013	0.119 ± 0.012	0.118 ± 0.017
Right Kidney	0.189 ± 0.016	0.165 ± 0.011	0.172 ± 0.012	0.165 ± 0.013
Left Kidney	0.187 ± 0.017	0.157 ± 0.012	0.170 ± 0.019	0.167 ± 0.017
Liver	1.321 ± 0.152	1.075 ± 0.126	1.207 ± 0.096	1.100 ± 0.081

All values are expressed as mean ± standard error and reported in g.

#### 3.1.4 Biochemical results.

**Fig 2:**
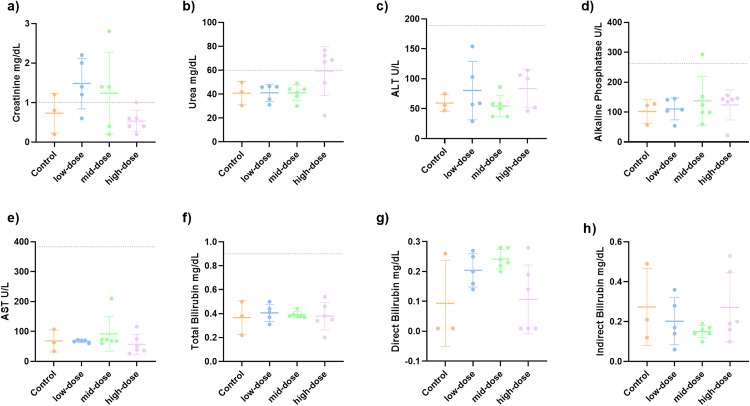
The biochemical markers in mice treated with CTX/PMB at three different CTX doses (low, medium, and high) and the control group (vehicle only) were quantified. The parameters included **A)** creatinine, **B)** urea, **C)** alanine aminotransferase (ALT), **D)** alkaline phosphatase, **E)** aspartate aminotransferase (AST), **F)** total bilirubin, **G)** direct bilirubin, and **H)** indirect bilirubin. The dashed lines indicate the upper reference limits for each parameter. Statistical analysis was performed via one-way ANOVA; no significant differences were found among the groups.

Biochemical analysis revealed no statistically significant differences among the groups for any of the evaluated parameters. However, mice treated with CTX/PMB at low and medium doses presented high mean levels of creatinine, which exceeded the upper reference limit (indicated by the dashed line). A slight increase in creatinine was also observed for one animal in the control group (Fig 2A). Additionally, the high-dose group presented high urea levels (Fig 2B). These variations suggested a response to PMB. All other biochemical parameters were within normal reference ranges (Fig 2C–2H).

### 3.2 Infection model

**Fig 3:**
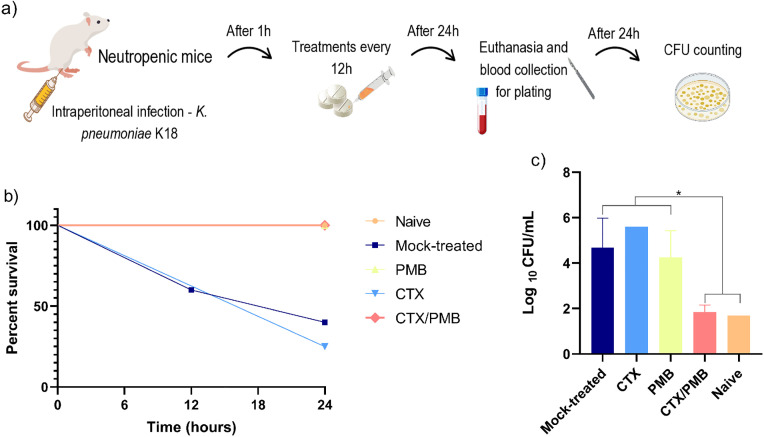
A) Schematic representation of the PC-R *K. pneumoniae* K18 strain infection model in mice used to evaluate the antimicrobial efficacy of the CTX/PMB combination against a resistant strain. **B)** Survival curves of mice treated with PMB, CTX, or the CTX/PMB combination following PC-R *K. pneumoniae* K18 infection. **C)** Bacterial load (log_10_ CFU/mL) across treatment groups. A lower detection limit of 1.69 log_10_ CFU/mL was established. Naïve (uninfected) and mock-treated (infected but untreated) control groups were included. Statistical analysis was performed via one-way ANOVA; the differences among groups were considered to be statistically significant at p < 0.05.

Based on previous evidence of the synergistic activity of CTX/PMB against PC-R *K. pneumoniae* and the results of the extended single-dose toxicity assay, a murine infection model was constructed to evaluate whether this effect is maintained in a complex mammalian *in vivo* system (Fig 3A). After 24 h, the CTX-treated mice exhibited a survival rate of only 25%, which was lower than that of the mock-treated group (survival rate of 40%). In contrast, all animals in the CTX/PMB-treated, PMB-treated, and naïve groups survived during this period (Fig 3B). The CTX/PMB-treated mice were more active throughout the experiment, suggesting higher treatment efficacy.

To determine the effectiveness of the treatments against PC-R *K. pneumoniae* K18 strain infection, the bacterial load was measured in mouse blood samples. The CTX/PMB regimen significantly decreased bacterial levels, reaching values comparable to those of the naïve (uninfected) group. However, mice treated with either antibiotic alone did not show the same improvement, with bacterial loads remaining similar to those in the mock-treated (infected and untreated) group (Fig 3C).

## 4. Discussion

The AMR has become a growing threat to global public health. To tackle this challenge, the continued development of effective therapies is crucial, with combination treatments emerging as cost-effective and timely solutions ^4^. CTX/PMB has shown strong synergistic activity against PC-R *K. pneumoniae*, demonstrated by a low fractional inhibitory concentration index (FICI < 0.5) and a high zero interaction potency (ZIP) synergy score of 37.484. The combination also showed no hemolytic activity (hemolysis rate < 5%) and no detectable toxicity in the alternative nematode model *Caenorhabditis elegans* [17]. These promising results encouraged this study to advance to preclinical testing in mice, as no such study has been reported to date, marking a vital step toward translating laboratory findings into clinically applicable treatment strategies.

The cyclic cationic polypeptide antibiotic, PMB, is considered an important last-resort treatment for MDR Gram-negative infections [26]. However, its clinical utility is limited by significant adverse effects, particularly neurotoxicity and nephrotoxicity, the latter often resulting in acute kidney injury characterized by reduced glomerular filtration rate and elevated serum levels of metabolic waste products such as creatinine and urea [29]. Interestingly, less toxic PMB derivatives are currently being study and might represent a promising solution for this problem [30,31]. The present study evaluated whether CTX, administered at three different doses in combination with a fixed dose of PMB, would influence or worsen the toxicity profile of PMB to develop safe CTX/PMB regimens.

Single-dose administration of CTX/PMB did not result in mortality or observable adverse effects in mice. Throughout 14 days of monitoring, all animals remained active and showed no signs of distress. Additionally, the combination treatment did not significantly affect body weight, food intake, or water consumption in the medium-dose or high-dose CTX groups. The maintenance of stable body weight in toxicity studies is considered to be an early indicator of general well-being, systemic safety, and preservation of organ integrity in preclinical models [32]. Therefore, the absence of weight loss in treated groups supports a preliminary safety profile of CTX/PMB.

After 14 days of monitoring, the mice were euthanized for organ weight assessment and biochemical analysis. The group treated with the higher dose of CTX in combination with PMB showed a significant reduction in the weight of the right kidney and liver, accompanied by an increase in plasma urea levels. The combination-treated groups also presented creatinine levels at the upper limit; however, a similar increase was noted in the control group, indicating that this effect may not be solely related to treatment. These results suggested a dose-dependent toxicity pattern associated with CTX, which may be amplified by the presence of PMB. Considering the well-known nephrotoxic profile of PMB, typically marked by increased serum levels of creatinine and urea [33], the findings of the present study highlight a possible pharmacotoxicological interaction of the combination on renal function at higher CTX doses, which should be carefully considered while developing clinical dosing regimens.

Polymyxins (PMB and colistin) exhibit neurotoxic effects through inhibition of acetylcholine release and mitochondrial toxicity. The neurotoxic effects of PMB are characterized by signs such as ataxia, convulsions, and pruritus [28,34]. The absence of these signs in the treated animals suggests that neurotoxic effects were not manifested at the administered dose of 2 mg/kg.

Based on the results and the indication of a dose-dependent toxic effect associated with CTX, the medium dose of CTX was selected for subsequent infection in an *in vivo* model. This was combined with PMB at 1 mg/kg/day, a dose selected according to its pharmacokinetic profile in the treatment of PC-R *K. pneumoniae* infections [28]. Compared to mono-therapies, CTX/PMB demonstrated enhanced antibacterial activity, significantly reducing the blood bacterial load of the PC-R *K. pneumoniae* in treated neutropenic mice while also prolonging overall survival and improving clinical conditions. Similar to the findings of the present study, other studies have shown that PMB exhibits synergistic effects with rifampicin against Gram-negative pathogens in neutropenic mouse models, reinforcing that PMB-based combination strategies may be effective in treating MDR infections [35,36].

From a clinical perspective, the translation of CTX/PMB therapy requires careful evaluation of dosing strategies to ensure optimal therapeutic outcomes. Given the pharmacokinetic properties of both agents, dose adjustment and therapeutic monitoring may help maintain efficacy while minimizing adverse effects (such as nephrotoxicity) during prolonged treatment, especially in critically ill patients [37,38]. Importantly, CTX/PMB represents a promising combination for clinical application, as both agents are already established as effective mono-therapies and are widely available for clinical use.

This study has some limitations. The toxicity assessment was based on a single-dose model, which may not reflect potential long-term effects, such as cumulative renal and neurological injury caused by antibiotics exposure. Further, the infection model used only one strain of PC-R *Klebsiella pneumoniae*, limiting the generalization of the findings to other strains or resistance profiles. Plus, the group sample size was small, which may have resulted in less robust statistical outcomes. The findings were based on a murine model, which may not fully reflect human responses. Finally, the observed increase in creatinine and urea suggests potential nephrotoxicity that requires a deeper investigation. Future studies should explore repeated-dose models, diverse bacterial strains, and advanced renal and neurotoxic assessments.

## 5. Conclusion

In conclusion, the CTX/PMB regimen demonstrated promising antibacterial efficacy against PC-R *K. pneumoniae* in a murine model, significantly reducing bacterial load and improving survival. These findings suggest that while CTX/PMB has therapeutic potential, careful optimization of dosing is essential to balance efficacy and safety, making further studies essential to establish the optimal therapeutic regimen and fully characterize the safety profile of this combination.

## References

[1] Ho CS, Wong CTH, Aung TT, Lakshminarayanan R, Mehta JS, Rauz S, et al. Antimicrobial resistance: a concise update. Lancet Microbe. 2025;6(1):100947. doi: 10.1016/j.lanmic.2024.07.010 39305919

[2] Wang M, Earley M, Chen L, Hanson BM, Yu Y, Liu Z, et al. Clinical outcomes and bacterial characteristics of carbapenem-resistant Klebsiella pneumoniae complex among patients from different global regions (CRACKLE-2): a prospective, multicentre, cohort study. Lancet Infect Dis. 2022;22(3):401–12. doi: 10.1016/S1473-3099(21)00399-6 34767753 PMC8882129

[3] Hasan SA, Raoof WM, Ahmed KK. First report of co-harboring bleomycin resistance gene (bleMBL) and carbapenemase resistance gene (blaNDM-1) Klebsiella pneumoniae in Iraq with comparison study among the sensitivity test, the BD Phoenix CPO detect test, and the Rapidec® Carba NP test. SJLSA. 2024;16(4):208–37. doi: 10.12731/2658-6649-2024-16-4-1249

[4] Aggarwal R, Mahajan P, Pandiya S, Bajaj A, Verma SK, Yadav P, et al. Antibiotic resistance: a global crisis, problems and solutions. Crit Rev Microbiol. 2024;50(5):896–921. doi: 10.1080/1040841X.2024.2313024 38381581

[5] Hasan SA, Raoof WM, Ahmed KK. Antibacterial activity of deer musk and Ziziphus spina-christi against carbapebem resis-tant gram negative bacteria isolated from patients with burns and wounds. Regul Mech Biosyst. 2024;15(2):267–78. doi: 10.15421/022439

[6] Coates ARM, Hu Y, Holt J, Yeh P. Antibiotic combination therapy against resistant bacterial infections: synergy, rejuvenation and resistance reduction. Expert Rev Anti Infect Ther. 2020;18(1):5–15. doi: 10.1080/14787210.2020.1705155 31847614

[7] Yu Y, Zhao H, Lin J, Li Z, Tian G, Yang YY, et al. Repurposing non-antibiotic drugs auranofin and pentamidine in combination to combat multidrug-resistant gram-negative bacteria. Int J Antimicrob Agents. 2022;59(5):106582. doi: 10.1016/j.ijantimicag.2022.106582 35378227

[8] Ataman M, Çelik BÖ. Investigation of the in vitro antimicrobial activity of eravacycline alone and in combination with various antibiotics against MDR Acinetobacter baumanni strains. BMC Microbiol. 2025;25(1):167. doi: 10.1186/s12866-025-03914-8 40133833 PMC11938564

[9] Petersen ME, Khamas AB, Østergaard LJ, Jørgensen NP, Meyer RL. Combination therapy delays antimicrobial resistance after adaptive laboratory evolution of Staphylococcus aureus. Antimicrob Agents Chemother. 2025;69(4):e0148324. doi: 10.1128/aac.01483-24 40084881 PMC11963546

[10] Xiao G, Li J, Sun Z. The combination of antibiotic and non-antibiotic compounds improves antibiotic efficacy against multidrug-resistant bacteria. Int J Mol Sci. 2023;24(20):15493. doi: 10.3390/ijms242015493 37895172 PMC10607837

[11] Chen M, Shao Y, Luo J, Yuan L, Wang M, Chen M, et al. Penicillin and cefotaxime resistance of quinolone-resistant Neisseria meningitidis clonal complex 4821, Shanghai, China, 1965-2020. Emerg Infect Dis. 2023;29(2):341–50. doi: 10.3201/eid2902.221066 36692352 PMC9881793

[12] El-Hariri SA, Saleh F, Moghnieh W, Sokhn ES. Phenotypic and molecular characterization of ESBL producing Escherichia coli and Klebsiella pneumoniae among Lebanese patients. JAC Antimicrob Resist. 2023;5(3):dlad074. doi: 10.1093/jacamr/dlad074 37305848 PMC10251202

[13] Araten AH, Brooks RS, Choi SDW, Esguerra LL, Savchyn D, Wu EJ, et al. Cephalosporin resistance, tolerance, and approaches to improve their activities. J Antibiot (Tokyo). 2024;77(3):135–46. doi: 10.1038/s41429-023-00687-y 38114565

[14] Readman JB, Acman M, Hamawandi A, Chiu C-H, Sharland M, Lindsay JA, et al. Cefotaxime/sulbactam plus gentamicin as a potential carbapenem- and amikacin-sparing first-line combination for neonatal sepsis in high ESBL prevalence settings. J Antimicrob Chemother. 2023;78(8):1882–90. doi: 10.1093/jac/dkad177 37283195 PMC10393883

[15] Bory C, Bory O, Guelfucci B, Nicollas R, Moreddu E. Deep cervical abscesses in children: efficacy of the cefotaxime-rifampicin combination. Eur J Pediatr. 2023;182(5):2315–24. doi: 10.1007/s00431-023-04917-1 36881146

[16] Ríos E, Pérez M, Sanz JC, Delgado-Iribarren A, Rodríguez-Avial I. Efficacy of delafloxacin alone and in combination with cefotaxime against cefotaxime non-susceptible invasive isolates of Streptococcus pneumoniae. Rev Esp Quimioter. 2024;37(2):158–62. doi: 10.37201/req/107.2023 38226580 PMC10945109

[17] Sturaro MC, Damaceno N da S, Faccin ID, Silva ON, de Aquino TM, Freire NML, et al. Synergistic antimicrobial combination of third-generation cephalosporins and polymyxin B against carbapenem-polymyxin-resistant Klebsiella pneumoniae: an in vitro and in vivo analysis. Antimicrob Agents Chemother. 2024;68(10):e0093024. doi: 10.1128/aac.00930-24 39254296 PMC11459926

[18] Fosse V, Oldoni E, Bietrix F, Budillon A, Daskalopoulos EP, Fratelli M, et al. Recommendations for robust and reproducible preclinical research in personalised medicine. BMC Med. 2023;21(1):14. doi: 10.1186/s12916-022-02719-0 36617553 PMC9826728

[19] Watabe T, Kaneda-Nakashima K, Ooe K, Liu Y, Kurimoto K, Murai T, et al. Extended single-dose toxicity study of [211At]NaAt in mice for the first-in-human clinical trial of targeted alpha therapy for differentiated thyroid cancer. Ann Nucl Med. 2021;35(6):702–18. doi: 10.1007/s12149-021-01612-9 33871803 PMC8134311

[20] Padda IS, Nagalli S. Cefotaxime. In: StatPearls. Treasure Island (FL): StatPearls Publishing; 2025. Available from: http://www.ncbi.nlm.nih.gov/books/NBK560653/32809488

[21] Hanafin PO, Kwa A, Zavascki AP, Sandri AM, Scheetz MH, Kubin CJ, et al. A population pharmacokinetic model of polymyxin B based on prospective clinical data to inform dosing in hospitalized patients. Clin Microbiol Infect. 2023;29(9):1174–81. doi: 10.1016/j.cmi.2023.05.018 37217076

[22] Oh S-H, Ahn J-S, Oh E-J, Kim Y-J, Yook J-M, Lim J-H, et al. Single-Dose Toxicity Study on ML171, a Selective NOX1 Inhibitor, in Mice. Biomed Res Int. 2021;2021:5515478. doi: 10.1155/2021/5515478 34195263 PMC8181097

[23] Sujjavorakul K, Katip W, Kerr SJ, Wacharachaisurapol N, Puthanakit T. Predicting the area under the plasma concentration-time curve (AUC) for first dose vancomycin using first-order pharmacokinetic equations. Antibiotics (Basel). 2023;12(4):630. doi: 10.3390/antibiotics12040630 37106993 PMC10135334

[24] Issuriya A, Cheaha D, Khan M, Panichayupakaranant P. Preclinical safety assessment and serum lipid profiles of wister rats following acute and subchronic exposure to water-soluble curcuminoid-rich extracts. ACS Omega. 2025;10(14):14271–82. doi: 10.1021/acsomega.5c00423 40256567 PMC12004289

[25] Gemeda HB, Debella A, Endale M, Abebe A, Mathewos M, Habtu W, et al. Acute and sub-acute toxicity of ethanol extracts of Hagenia abyssinica and Rumex abyssinicus flowers in Swiss albino mice. PLoS One. 2025;20(2):e0319464. doi: 10.1371/journal.pone.0319464 39999122 PMC11856396

[26] da Silva KE, Thi Nguyen TN, Boinett CJ, Baker S, Simionatto S. Molecular and epidemiological surveillance of polymyxin-resistant Klebsiella pneumoniae strains isolated from Brazil with multiple mgrB gene mutations. Int J Med Microbiol. 2020;310(7):151448. doi: 10.1016/j.ijmm.2020.151448 33092694

[27] Sturaro MC, de Souza GH de A, Damaceno N da S, Silva ON, de Aquino TM, Freire NML, et al. Antimicrobial activity of ceftibuten/polymyxin B combination against polymyxin/carbapenem-resistant Klebsiella pneumoniae. J Antimicrob Chemother. 2025;80(1):116–25. doi: 10.1093/jac/dkae382 39450857

[28] Yu Z, Liu X, Du X, Chen H, Zhao F, Zhou Z, et al. Pharmacokinetics/pharmacodynamics of polymyxin B in patients with bloodstream infection caused by carbapenem-resistant Klebsiella pneumoniae. Front Pharmacol. 2022;13:975066. doi: 10.3389/fphar.2022.975066 36588676 PMC9800617

[29] Liu L, Liu Y, Xin Y, Liu Y, Gao Y, Yu K, et al. An early and stable mouse model of polymyxin-induced acute kidney injury. Intensive Care Med Exp. 2024;12(1):88. doi: 10.1186/s40635-024-00667-y 39352603 PMC11445218

[30] Tang H, Zhang Y, Ma J, Dong Y, Gao Q, Feng J. Design, synthesis and antimicrobial studies of some polymyxin analogues. J Antibiot (Tokyo). 2020;73(3):158–66. doi: 10.1038/s41429-019-0262-0 31831870

[31] Kim SJ, Jo J, Kim J, Ko KS, Lee W. Polymyxin B nonapeptide potentiates the eradication of Gram-negative bacterial persisters. Microbiol Spectr. 2024;12(4):e0368723. doi: 10.1128/spectrum.03687-23 38391225 PMC10986493

[32] Silva AV, Norinder U, Liiv E, Platzack B, Öberg M, Törnqvist E. Associations between clinical signs and pathological findings in toxicity testing. ALTEX. 2021;38(2):198–214. doi: 10.14573/altex.2003311 33118607

[33] Hudson CS, Roy A, Li Q, Joshi AS, Yin T, Kumar A, et al. Mechanisms of gelofusine protection in an in vitro model of polymyxin B-associated renal injury. Am J Physiol Renal Physiol. 2024;327(1):F137–45. doi: 10.1152/ajprenal.00029.2024 38779756 PMC12180518

[34] Radkowski P, Oszytko J, Sobolewski K, Trachte F, Onichimowski D, Majewska M. The effect of antibiotics on the nervous system: importance for anesthesiology and intensive care. Antibiotics. 2025;14(6):622. doi: 10.3390/antibiotics14060622 40558212 PMC12189792

[35] Zhang H, Zhu Y, Yang N, Kong Q, Zheng Y, Lv N, et al. In vitro and in vivo activity of combinations of polymyxin B with other antimicrobials against carbapenem-resistant Acinetobacter baumannii. Infect Drug Resist. 2021;14:4657–66. doi: 10.2147/IDR.S334200 34764660 PMC8577563

[36] Van Den Berg S, Sassen SDT, Couet W, Marchand S, Van Der Spek H, Ten Kate MT, et al. The synergistic effect of the combination of polymyxin B and rifampicin in a murine neutropenic thigh infection model with E. coli and K. pneumoniae. J Antimicrob Chemother. 2025;80(5):1248–55. doi: 10.1093/jac/dkaf056 40036260 PMC12046403

[37] Li Y, Deng Y, Zhu Z-Y, Liu Y-P, Xu P, Li X, et al. Population pharmacokinetics of polymyxin B and dosage optimization in renal transplant patients. Front Pharmacol. 2021;12:727170. doi: 10.3389/fphar.2021.727170 34512352 PMC8424097

[38] Dillies T, Perinel-Ragey S, Correia P, Morel J, Thiery G, Launay M. Dosing regimen for cefotaxime should be adapted to the stage of renal dysfunction in critically ill adult patients-a retrospective study. Antibiotics (Basel). 2024;13(4):313. doi: 10.3390/antibiotics13040313 38666989 PMC11047316

